# Domestic Medicine

**Published:** 1871-10

**Authors:** 


### Domestic Medicine.

### USEFUL RECIPES FOR THE HOUSEHOLD.

SPOTS ON MAHOGANY.—Stains and spots may be taken out of mahogany with a little aqua-fortis or oxalic acid and water, rubbing the part by means of cork, till the color is restored, observing afterwards to wash the wood well with water, and to dry and polish as usual.

---

FOR COUGH.—Roast a lemon very carefully without burning it; when it is thoroughly hot, cut and squeeze into a cup upon three ounces of sugar, finely powdered. Take a spoonful whenever your cough troubles you. It is good and agreeable to the taste. Rarely has it been known to fail of giving relief.

---

HOW TO COOK BEETS.—Beets should be carefully washed, but not cut before boiling, as cutting them allows the juice to escape, leaving them white and hard. In summer, boil them an hour in salted water, and in winter, boil them four hours. After boiling, scrape off their skins and cut off the threads hanging from them.

---

TO COOK BIRDS FOR CONVALESCENTS.—Lay them upon the gridiron; broil until they have a light brown color; then put them in a stewpan; pour over hot water enough to cover them. Let them stew until tender. Season with a little fresh butter, pepper and salt. Chicken, birds and squirrels, stewed in a double kettle, are very delicate for invalids. If permitted, stuff the fowls and birds with minced oysters.

---

HOW TO BROIL WITHOUT BURNING.—In broiling a beef-steak, whenever the coals blaze up from the drippings, a pinch of fine salt thrown upon them will instantly extinguish the flames. By carefully attending to this matter, you may have your broiled steak or chicken, crisp, but not scorched, and juicy, yet well done.

---

TO REMOVE WARTS.—Dissolve an ounce of white vitriol in five table-spoonfuls of water, put into a vial, and rub the warts three or four times a day and oftener if convenient. In two weeks they will be gone, without pain or scar. Other remedies are to moisten the tops of the warts once a day with creosote; or burn a piece of linen or cotton on any piece of steel, and rub the moisture left by burning, on the warts, repeating the operation three or four times.

DIARRHŒA IN CHILDREN.—Blackberry cordial is very useful in bowel complaints of children. The cordial is easily made in the following manner:

Take five quarts of blackberry juice, two quarts of white sugar syrup, and one quart of good brandy. Pour the juice and syrup together, heat to the boiling point, and when cool, add the brandy. Bottle tightly, when it will be ready for use. Dose from one teaspoonful to a tablespoonful, three times daily.

---

PASTE FOR LABELLING ON TIN.—Any paste, such as you buy in commerce, or make yourself from gum-arabic or gum-tragacanth, with the addition of a little wintergreen oil, will do for this purpose. It is only necessary to remove from the tin a thin film of grease which prevents perfect adhesion, causing it to blister off on drying. This is accomplished by dipping a rag in a dilute solution of caustic soda or potash—ten of water to one of potash—rubbing the spot on which to fix the label, and drying it with another rag. No label put on in this way will come off again.

---

DARKNESS IN TREATMENT OF SMALL-POX.—Mr. J. H. Waters states, that if a patient, in the beginning of the attack, be put in a room from which absolutely all light is excluded, save that of a candle, the effect is to arrest the disease in the papular or vesicular stage; it never becomes purulent, and the skin between the vesicles is never inflamed or swollen; the liquor sanguinis is prevented from becoming pus; the large scabs of matter never form over the face; there is no intense pain, and only trifling itching, and the smell is either very slight or altogether wanting.—*London Lancet.*

---

FEEDING BOTTLES, FOR BABIES.—All feeding bottles that are so constructed as to require india-rubber tubing or nipples, are dangerous.—The suction of the child always open the pores of the rubber, into which more or less milk escapes, and is confined there, when the suction is removed and the pores collapsed. The result is that this milk soon becomes sour and putrid, spoiling the sweet milk with which it comes in contact. The caoutchouc, or rubber, contains sufficient zinc or white lead to produce poisonous effects. Many fatal cases of poisoning of infants have been reported, the cause having been traced to the use of these bottles.

**TO TAKE BRUISES OUT OF FURNITURE.**—Wet the part with warm water; double a piece of brown paper [illegible] or six times, soak it in the warm wat[illegible] [illegible]y it on the place; apply on that a wa[illegible] but not hot flat-iron, till the moisture is evaporated. If the bruise be not gone, repeat the process. After two or three applications, the dent or bruise will be raised to the surface. If the bruise be small, merely soak it with warm water, and hold a red-hot iron near the surface, keeping the surface continually wet—the bruise will soon disappear.

---

**AIR YOUR BEDS.**—The wise housekeeper will see to it that all the beds should be aired immediately after occupancy. The impurities which emanate from the human body, from insensible perspiration, are made of minute atoms, which, if allowed to remain long, are absorbed by the bed, and will then, to a greater or less extent, vitiate the air for a considerable time afterward. Let the occupant throw the bed open upon rising, and as soon as is convenient, open the window and ventilate the sleeping room. One hour's early ventilation is worth two hours' late airing.

---

**SWEET PICKLES.**—The following is a good recipe for making sweet pickles of peaches, tomatoes, apples, etc. For each nine pounds of fruit, take three pounds of sugar, one pint of vinegar and one-half ounce of cloves. Put the sugar and vinegar together in a preserving kettle, let them come to a boil, then put in cloves, ground, if for apples; if for peaches or tomatoes, put two whole cloves in each, or more as you like. Put your fruit into the syrup, let it boil until it cracks open, then lift it out carefully, boil down the juice, and pour it over them. As the juice gets thinner by standing, drain it off, and boil it down [illegible]s much as you conveniently, pouring it [illegible]ver the fruit again.

---

**TO POLISH MARBLE, ETC.**—Marble of any kind, alabaster, any hard stone or glass may be repolished by rubbing it with a linen cloth dressed with oxide of tin (sold under the name of putty powder). For this purpose a couple or more folds of linen should be fastened tight over a piece of wood, mat or otherwise, according to the form of the stone. To repolish a mantel-piece it should be first perfectly cleaned. This is best done by making a paste of lime, soda and water, well wetting the marble, and applying the paste. Then let it remain a day or so, keeping it moist during the interval. When this paste has been removed, the polishing may begin. The linen and putty powder must be kept constantly wet. Glass, such as jewelers' show-counter cases, which becomes scratched, may be polished in the same way.

---

**LAUNDRY POLISH FOR LINEN.**—Add to starch made in the usual way, a small lump of white sugar, or a bit of white wax or spermaceti, or a few thin shavings of white soap, and a teaspoonful of salt. After the clothes are rinsed in the blue water, starch them, and dry on the clothes-line; then wring them from cold water, roll up tightly, and let them lie awhile. Iron smoothly in the usual way. Then place the bosom or piece to be polished, on a board with a single fold of muslin over it, pass a damp cloth over the linen and polish with an iron made for that purpose, such as may be bought at the hardware or kitchen furnishing stores.

---

**SICKNESS FROM WALL PAPER.**—We have before warned our readers against pasting severa thicknesses of paper upon the wall. It is doubt less, a prevalent cause for malignant fevers in most of our larger cities. A Barracks in London being very offensive, and the authorities being unable to devine the cause, and the establishment being threatened with fever, a thorough investigation was made of the place, which led to the taking up of the drains, tearing away of the floors, &c., without, however, revealing the cause of the smell. Finally, in tearing down the walls, several thicknesses of wall paper were discovered, and pasted over one another, until a thickness amounting to fourteen layers was found. Between these layers, and reveling in rotten paste, were millions of worms, and fungi. This at once revealed both the cause of the foul air and of the sickness.

---

**SUNSHINE.**—The time will very likely come when sunshine, or sunlight, will be so utilized as to be the entire remedy used for very many diseases. That it is a wonderful vitalizer, none can doubt who know anything about it.

But how many houses are so constructed with a view to getting all the sunshine possible, especially when so much needed as in winter and spring? The living, or sitting-room, at these seasons of the year, at least, should have a full southern exposure, with large windows to let in the sunshine. Sleeping rooms, wardrobes, closets passage-ways, should receive the cleansing, verifying influence of the sun. Sickly persons should court the sunshine as much as possible,—sit in it, lie in it, luxuriate in it. It doesn't cost anything, only appreciation.

A room warmed neither by the sun nor by fire is unhealthy, and not fit for human habitation. It is a poor theory that sends men, women or children off into a cold room to sleep, on health principles, when warmth has been excluded for a day or a week, or perhaps months. The change in the temperature of a room, having both fire and sunshine, after the sun goes down, is exceedingly marked. A perceptible chill is felt.

---

TO AVOID AGUE.—The first suggestion, of course, is to leave those districts where this troublesome complaint prevails. Sometimes, however, one's residence cannot well be changed. To persons so circumstanced, there are preventions by the use of which the majority might generally escape it, which are referred to in the *Journal of Health*, as follows:

1. Avoid exposing themselves to the malarial air after sunset and before sunrise. 2. Occupy rooms at night on the sunny side of the house and up stairs. 3. Build a fire in the house as soon as the dew begins to fall. The heat of the fire will do much to kill the malaria. 4. Keep the skin healthy and active by a thorough bath every day on rising, in a warm room, with sufficient friction to produce a healthy reaction. 5. Keep the bowels open by a proper diet. In nine cases out of ten, the cause of the ague would be easily overcome if the depurating organs were not overtaxed and morbid matters allowed to accumulate in the system to oppress it.

---

THE Suez Canal is rapidly filling up with drifting sands. Vessels of heavy tonnage already find diffilculty in making the transit on this account. It is to be hoped that the indomitable spirit that projected and executed this, an undertaking so vast and useful, will yet find some availabe means to protect it from the drifting sands that allready threaten to render this great maratime acheivement of non effect.

---

INFALLIBLE REMEDIES.—For corns, easy shoes; for bile, exercise; for rheumatism, new flannel and patience; for gout, toast and water; for the toothache, a dentist; for debt, industry; and for love, matrimony.

